# Annotation

**Published:** 1954-07

**Authors:** 


### *Annotation*

### BUNGALOWS FOR DISABLED PERSONS

The Urban District Council of Burnham-on-Sea has provided a group of bungalows specially designed for disabled persons. The following notes give a brief description of these:

1. They have been primarily designed on the assumption that the disablement necessitates the person using a wheel-chair.
2. There are no steps in the approach to the bungalow, ramps being provided.
3. A verandah outside the entrance gives a convenient place in which to leave the wheel-chair.
4. The entrance hall is very wide, enabling a wheel-chair to use the hall readily and to turn around within its width.
5. All doors are of sufficient width to allow the passage of a wheel-chair.
6. Three bedrooms are provided, but as a disabled person might work at home it is understood one bedroom can be used as a workroom.
7. The water-closet compartment is wide and is provided with chromium hand-grips, one on each side of the pan.
8. The bathroom is provided with fittings which the disabled person can use to help himself into the bath.
9. In one bedroom a hand-grip is provided which the disabled person can use to help himself into bed.

